# Healthcare system complexity of hepatitis B and C related liver disease: service mapping in New South Wales, Australia

**DOI:** 10.3389/frhs.2026.1842589

**Published:** 2026-06-25

**Authors:** Shenghan Cai, Karen Hutchinson, Amany Zekry, Rose Boutros, Milena Lewandowska, Stephen Goodall, Jacob George, Yvonne Zurynski

**Affiliations:** 1 Centre for Healthcare Resilience and Implementation Science, Australian Institute of Health Innovation, Faculty of Medicine, Health and Human Sciences, Macquarie University, Macquarie Park, NSW, Australia; 2 Central Coast Research Institute for Integrated Care, Gosford, NSW, Australia; 3 School of Population Health, Faculty of Medicine and Health, The University of New South Wales, Kensington, NSW, Australia; 4 School of Clinical Medicine, St. George and Sutherland Clinical Campus, The University of New South Wales, Kensington, NSW, Australia; 5 Department of Gastroenterology and Hepatology, St. George Hospital, Kogarah, NSW, Australia; 6 Storr Liver Centre, The Westmead Institute for Medical Research, Westmead, NSW, Australia; 7 Sydney Medical School, The University of Sydney, Camperdown, NSW, Australia; 8 Centre for Health Economics Research and Evaluation, the University of Technology Sydney, Ultimo, NSW, Australia; 9 Department of Gastroenterology and Hepatology, Westmead Hospital and Western Sydney Local Health District, North Parramatta, NSW, Australia

**Keywords:** barriers and facilitators, healthcare system complexity, hepatocellular carcinoma, implementation, integrated care, person-centred care, primary prevention, viral hepatitis B and C

## Abstract

**Background:**

Early detection and treatment of hepatitis B and C virus (HBV/HCV) infection, along with regular monitoring for hepatocellular carcinoma (HCC), are critical strategies for improving health outcomes and reducing disease burden. An integrated care pathway should encompass person-centred approaches to prevention, diagnosis, treatment, and surveillance for HCC. Despite global efforts in hepatitis control, how hepatitis and liver cancer services connect at a population level remains unclear.

**Methods:**

Semi-structured interviews were conducted with 42 public health staff, nurses, and medical specialists (February 2023 to December 2024). Participants represented hepatitis and hepatology/gastroenterology clinical services across all 15 Local Health Districts in New South Wales (NSW), Australia. Inductive thematic analysis explored perceptions of service integration and person-centred care for hepatitis and HCC. A service mapping exercise was conducted to explore the population-level landscape of hepatitis-HCC service delivery, identifying key stakeholders, resources, care pathways, and models of care.

**Results:**

The study identified nine strategic/system-level stakeholders, including HIV and Related Program units, Public Health Units, Specialist Liver Services, primary care and community services. Participants described hepatitis-HCC care journeys and pathways that were adapted to varied local level contexts, populations and resources. Three dominant service models emerged: (1) rural service model, (2) integrated care model, (3) integrated multidisciplinary partnership model. Despite fragmented pathways, complex care needs, and ongoing resource constraints, notable innovations were described, including nurse-led outreach clinics in the community, and multidisciplinary liver cancer teams offering tele-mentoring to rural clinicians. Five interrelated system-level challenges emerged: (1) inadequate and insecure workforce, (2) fragmented care pathways, (3) misaligned funding, (4) inconsistent data and information systems, and (5) stigma and limited community awareness.

**Conclusions:**

This is the first jurisdiction-wide mapping of hepatitis-HCC service pathways in NSW. The findings offer actionable insights to strengthen service integration, promote person-centred care continuity, and inform strategic planning for hepatitis and liver cancer services. Supporting these efforts can transform isolated innovations into cohesive, equitable liver health services, accelerating progress toward World Health Organization's hepatitis elimination targets and serving as a scalable model for integrated liver care globally.

## Introduction

Hepatocellular carcinoma (HCC) is the most prevalent primary liver cancer among the global adult population, with over 70% of cases estimated to be associated with chronic hepatitis B/C virus (HBV/HCV) infections (1). In addition, cases of HCC linked to metabolic syndrome are increasing (2). With a five-year survival rate of less than 20% (3), HCC has profound impacts on patients’ physical, psychological, and social wellbeing (4).

Early detection and treatment of HBV and HCV infections, and regular monitoring for liver disease (e.g., liver fibrosis, cirrhosis, and HCC) are proven to be vital and cost-effective in improving individual outcomes (5–7). However, many people living with chronic HBV/HCV are without noticeable or recognisable symptoms (8). They may go undiagnosed for years, and present late for care with cirrhosis (6, 9), or with advanced HCC by which time curative treatment options are limited (3, 4, 10). Programs for primary prevention of HBV/HCV are mainly targeted towards individuals at high risk of acquisition, transmission, or morbidity (11–13), leaving out populations who may have HBV/HCV but are not identified as being at high risk.

The development of effective antiviral therapy for HBV and HCV infections has prompted the World Health Organization (WHO) to set a global elimination target for hepatitis as a public health concern by 2030 (11–13). In response, prevention policies and interventions have centred on education and awareness, vaccination, screening, treatment, and linkage to care for disease surveillance (14–16). Despite global elimination goals, few studies have systematically mapped how prevention, treatment, and surveillance services interconnect at a population level (7, 12). In theory, pathways should be implemented to support the continuum of care that spans HBV/HCV testing, treatment uptake and adherence leading to viral suppression or sustained virologic response (17), alongside surveillance for the early detection and management of liver fibrosis and cirrhosis (18). However, in practice, the integration between primary prevention, early treatment, and the long-term reduction in liver disease burden, such as fibrosis, cirrhosis, and HCC, remains poorly understood. Few studies have described successful service models that include integrated pathways that support the continuum of care from hepatitis to HCC. Available evidence is mostly based on single site case studies, often focused on specific migrant communities or drug and alcohol clinics (14–16, 19). To improve the continuum of care and hepatitis-related liver care pathways, we need a clearer understanding of how these systems are currently implemented.

This study aimed to map care pathways and the key services and organisations involved in the prevention and treatment of HCC in NSW, Australia. It investigates service gaps and the barriers and facilitators shaping service integration to inform improvements for the hepatitis-HCC care continuum in NSW.

### Study context

Australia has a complex health system with multiple government and non-government agencies and organisations responsible for care delivery (20). Hepatitis-related and government-funded clinical care and services are mostly covered under the Australian universal healthcare scheme, Medicare (21). Equitable access to government-funded direct-acting antiviral therapy for HCV has been available since 2016 (22). A wide range of healthcare professionals (HCPs), including gastroenterologists, hepatologists, general practitioners (GPs), nurse practitioners, drug and alcohol specialists, and sexual health specialists, can initiate treatment for HCV infections. While there is no cure for HBV, free vaccination is available through the National Immunisation Program for newborns, unvaccinated children, and high-risk adults (23). HBV antiviral treatments are subsidised under the Pharmaceutical Benefits Scheme and can be prescribed by HCPs, including GPs, after completing training under the Section 100 Highly Specialised Drugs Program (24).

In New South Wales (NSW), 15 Local Health Districts (LHDs) manage community health promotion, resource allocation, and public hospital services under the NSW Ministry of Health (25, 26). Primary care is funded at the national level and delivered chiefly by GPs, with Primary Health Networks (PHNs) providing GP education, support, and locally commissioned health programs (27). Target groups for hepatitis prevention and care are people living with socioeconomic disadvantage, including unstable housing, migrants or refugees from HBV/HCV endemic regions, Aboriginal and Torres Strait Islander peoples (First Nations), older adults, and people with mental health challenges (13, 28–30). Prevention programs also target groups engaging in risky behaviours, (e.g., high-risk sexual practices, sex workers, people who inject drugs) (12, 28–30).

The population of NSW is predominantly concentrated in eastern urban and coastal areas, with 61% residing in the Greater Sydney Area (31). The imbalance in population distribution across urban and rural regions results in inequities in available resources, employment opportunities, and access to public health services in rural and remote areas, limiting timely access to prevention and care (32–35).

## Methods

### Ethics and project governance

Ethics approval was granted by the Macquarie University Human Research Ethics Committee (reference no. 520241259558948). This study is part of the Accelerated translational research in Primary Liver Cancer (APRICA) program, which aims to improve liver cancer outcomes in NSW. The NSW Ministry of Health helped to promote the study, but was not involved in the study design, data collection, analysis, or interpretation. A multi-disciplinary project steering committee, including people living with hepatitis, provided guidance.

### Study design

This mixed-methods study employed semi-structured in-depth interviews and service landscape mapping, while leveraging the Consolidated Framework for Implementation Research (CFIR) domains as an organising framework (36). Interviews were conducted with key informants from services and organisations involved in HBV/HCV prevention and treatment, liver disease care, and HCC management. The service mapping was developed through web searches and information gathered during interviews and consultations. A purposive sampling frame was adopted to include views of multiple professionals (e.g., program officers, unit managers, specialist nurses, and medical specialists from local liver, hepatology, gastroenterology, or oncology services based in publicly funded hospitals) across all 15 NSW LHDs. The reporting of this study follows the Consolidated Criteria for Reporting Qualitative Research (COREQ) guidelines (37).

### Recruitment and participants

A list of potential interviewees was generated based on publicly available information from the NSW Ministry of Health website and web searches to identify key hepatitis prevention programs and liver cancer clinical care services. An email invitation with a participant information and consent form was circulated to 17 Public Health Units (PHUs), 12 HIV (Human Immunodeficiency Virus) and Related Programs (HARP) units and 10 public hospitals with specialist liver services across the 15 LHDs. In some regions, a single HARP unit operated across multiple LHDs, and a single LHD could have two to three PHUs. A snowballing approach was used, where people interviewed recommended others with key, relevant roles. After 2 weeks, non-responders were followed up twice, 2 weeks apart.

A total of 24 service leaders responsible for hepatitis and liver-related services were identified across the 17 PHUs and 12 HARP units, noting that some leaders oversaw multiple units across different LHDs. Of these, 17 staff members agreed to participate, with one participant later withdrawing due to personal reasons. Across the 10 public hospitals with specialist liver services, 31 clinical leaders were identified, and 26 agreed to participate in interviews. A total of 16 staff from all 15 LHDs contributed to the study, including representatives from 11 of 12 HARPs (12 staff) and 4 of 17 PHUs (2 PHU staff and 2 Sexual Health/D&A staff delegated by their PHUs). Nurses (*n* = 11) and liver specialists (*n* = 15) from eight of the 10 specialist liver services also participated. Some participants held multiple roles across public health and clinical settings, including dual appointments within LHDs (e.g., two staff worked across PHUs and HARP units) and combined clinical and program roles in hepatitis and chronic liver disease services (e.g., three nurses worked in both, clinics and HARP units).

Both written and verbal consent was obtained. Participants were offered no incentives or compensation and were informed that there would be no consequences if they opted not to participate or dropped out of the study at any stage. Services for individuals confined within correctional facilities were excluded due to their distinct context and methodological requirements beyond the scope of this study. In contrast, community correction services were included, as they support people living under strict supervision in the community and form part of the service population benefiting from LHD public health services.

### Data collection

Two semi-structured interview guides were developed, piloted, and revised based on feedback from public health and clinical experts (Supplementary Files S1, S2). Key topics included: local high-risk groups, population characteristics, availability of local services, prevention programs and strategies, integration with hepatitis and HCC services pathways and models, and experiences of delivering care or programs, including perceived challenges and enablers. Interviews were conducted on Microsoft Teams (MS-Teams) by three trained and experienced qualitative researchers (YZ, SC, KH) between February 2023 and December 2024. Interviews were recorded, transcribed verbatim, and anonymised before analysis (SC). To avoid identifying individuals, many of whom held unique roles, LHDs were also de-identified. In addition, consultations with PHNs (*n* = 9, YZ) and Australasian Society for HIV, Viral Hepatitis and Sexual Health Medicine (ASHM) (*n* = 3, KH, SC) were carried out via emails or brief MS-Teams discussions to understand the HBV/HCV prevention strategies and programs in primary care settings; meetings were documented through minutes or notes written during and immediately after the consultations (YZ, SC, KH).

In addition, a landscape mapping exercise was undertaken to understand the key organisations involved in the prevention, detection, treatment and management of HBV/HCV, surveillance and treatment of related liver disease—fibrosis, cirrhosis, and HCC across NSW. Through an initial consultation with clinical experts involved in the APRICA project and key informants from the NSW Ministry of Health, including HARPs and PHU staff, we developed a preliminary roadmap of hepatitis, chronic liver disease, and HCC service components. As interviews commenced, it became evident that LHDs organise these components in markedly different ways, with varying levels of integration—patterns that had not previously been documented. This variation highlighted the importance of systematic service mapping in providing essential contextual grounding for interpreting interview data and for understanding the different enablers and challenges across LHD liver health services.

### Data analysis

Interview transcripts were analysed as they became available to identify broad candidate themes. Thematic saturation was reached after 10 interviews with staff from PHUs and HARP units and after 12 interviews with HCPs working across LHDs. We continued with additional interviews to capture local service variations across the LHDs. Two researchers (SC, KH) familiarised themselves with the data and conducted initial coding independently on four interview transcripts using an inductive thematic approach, with no predetermined theoretical frameworks and open coding directly informed by the raw data (38). Initial codes were generated based on the emerging patterns and themes expressed by participants. A coding framework was collaboratively developed and iteratively refined, and discrepancies were resolved through team discussion. The coding framework was then applied to code each transcript in NVivo 14 (QSR International, 2023) by two researchers (SC, KH).

A table describing key stakeholder organisations was developed, and rich pictures (39) were created from the service mapping data to visually depict the models of care operating across NSW as described by key informants. Rich pictures are visual diagrams that capture the multiple interacting components of a complex system (39), helping to illustrate the interconnected services and pathways involved in liver care. Information from reports and policy documents provided contextual background to the service descriptions provided by participants. Special attention was given to how services are coordinated and connected across primary prevention programs, community-based care, specialist liver services, and disease surveillance programs.

A member checking process was undertaken to refine and validate the findings. After preliminary analysis, early themes and service mapping outputs were shared with participants. To maintain anonymity, only staff who had contributed LHD-specific service mapping data were invited to review the rich pictures for their respective LHD. Feedback was gathered through follow-up discussions via email or online meetings to confirm accuracy, ensure anonymity, and assess the resonance of emerging interpretations.

### Reflexivity statement for researchers

The three researchers who conducted the interviews and service mapping were experienced health services researchers who were not involved in hepatitis or liver cancer services or clinical care, though all had personal experience navigating the Australian healthcare system. YZ is a senior academic and health services researcher with over 30 years of experience in health research and approximately 15 years of experience in mixed methods studies, integrated models of care, and health services research. KH is an allied health clinician and senior health services researcher with experience in qualitative studies and implementation research. Her background as a physiotherapist with many years of clinical experience supporting people living with complex neurological conditions and accessing multiple health services, enabled a deeper understanding of services, referral pathways and service integration. SC is a demographer and anthropologist with more than 10 years of experience in mixed-methods public health research experience across multiple health systems, with a focus on equity of care. Her cultural background differed from many participants, prompting reflexive consideration of how cultural positioning might shape data collection and interpretation. The wider team, although not directly involved in data collection and analysis, contributed methodological and clinical perspectives. Throughout the study, the team engaged in ongoing reflexive practice to consider how their backgrounds and assumptions might shape data collection and analysis.

## Results

### Characteristics of participants and key stakeholders

Forty-two key informants were recruited across all 15 NSW LHDs; 60% were female and 40% male; 45% worked in metropolitan areas and 55% in regional NSW (Table 1).

**Table 1:** Characteristics of participants.

Roles	Number of participants *N* (%)	Service areas
Nurses (dedicated to hepatitis/liver services)	11 (26%)	4 metropolitan, 7 rural/regional
Specialists (e.g., Hepatologists, Gastroenterologists, Oncologists)	15 (36%)	8 metropolitan, 7 rural/regional
Staff members (from HARP, PHUs, D&A, Sexual Health, etc.)	16 (38%)	7 metropolitan, 9 rural/regional

Some participants held multiple roles, only their primary role related to prevention or treatment of hepatitis, liver disease, or HCC is reported. One participant withdrew prior to interview for reasons unrelated to the study and was not included in the analysis. HARP, HIV and related programs units; PHUs, public health units; D&A, drug and alcohol services, also known as AOD (Alcohol and Other Drugs) in some LHDs.

The stakeholder mapping exercise identified many different organisations or organisational units that are involved in developing policy and delivering education, prevention programs, and clinical services related to HBV/HCV prevention, detection, and treatment, as well as liver disease and HCC monitoring, diagnosis and treatment (Table 2).

**Table 2:** NSW key stakeholder organisations for hepatitis and liver disease prevention and care.

Organisation	Primary functions	Funding
Local Health Districts (LHDs)	Administrative entities responsible for promoting the health of the local community managing health resources delivering public health services through hospitals, emergency departments, and outpatients’ clinics including liver clinics and services Each LHD responsible for managing *public hospitals* and health institutions and for providing health services to defined geographical areas of NSW. Each LHD also include *public health services entities* such as HIV (human immunodeficiency virus) and other related programs (HARP), Drugs and Alcohol (D&A), Sexual Health, Public Health Unit (PHU), etc.	NSW Ministry of Health Australian Government
Primary Health Networks (PHNs)	Independent organisations that manage health regions for improving the efficiency and effectiveness of health services for people, particularly those at risk of poor health outcomes improving the coordination of health services increasing access and quality support for people	Australian Government
Cancer Institute NSW	A pillar organisation of NSW Health providing the strategic direction for cancer control in NSW aims to: reduce cancer incidence in the community increase survival rates of people diagnosed with cancer improve the quality of life for people with cancer and their carers provide a source of expertise on cancer control for the government, health service providers, medical researchers and the general community data custodian of the NSW Cancer Registry	NSW Ministry of Health
Aboriginal Community Controlled Health Services (ACCHS)/Aboriginal Medical Services (AMS)	The voice on Aboriginal Health and representing Aboriginal community-controlled health organisations addresses the needs of Aboriginal people to improve Aboriginal health outcomes delivers holistic and responsive health and medical care that includes, social, emotional, physical and cultural wellbeing of individuals, families and communities	NSW Ministry of Health Australian Government
Gastroenterological Society of Australia (GESA)	A peak membership organisation for Australian healthcare professionals and researchers working in the fields of gastroenterology and hepatology promotes and continuously improves the standards of clinical practice in gastroenterology and hepatology in Australia provides clinical guidelines, mentorship, and expert advice on treatment and case management for HBV, HCV, and chronic liver disease	N/A
Australasian Society for HIV, Viral Hepatitis and Sexual Health Medicine (ASHM)	A peak organisation of health professionals in Australia and New Zealand conducts work in HIV, viral hepatitis, other blood-borne viruses and sexually transmissible infections generates knowledge and action in clinical management and research, education, policy and advocacy in Australasia and internationally	Australian Government
Hepatitis NSW (Hep NSW)	A not-for-profit charity for improving the health and wellbeing of communities affected by hepatitis B and C	NSW Ministry of Health
AIDS Council of NSW (ACON)	NSW's leading HIV and LGBTQ + health organisation providing community health, inclusion and HIV responses for people of diverse sexualities and genders.	Australian Government NSW Ministry of Health
NSW Users and AIDS Association (NUAA)	A not-for-profit NSW peer-based organisation advocates for people who use drugs, particularly those who inject drugs advocates for services supporting the diversity of people impacted by stigma and discrimination caused by the criminalisation of drug use across NSW	NSW Ministry of Health

Main stakeholder organisations only, as the list is not exhaustive and there may be other important organisations working in other specific contexts. Children's services and incarceration settings are excluded. HBV, hepatitis B virus; HCV, hepatitis C virus; HIV, Human Immunodeficiency Virus. All organisational descriptions were drawn directly from each body's official website: LHD details from the NSW Health LHD Directory (https://www.health.nsw.gov.au/lhd/Pages/default.aspx) and the NSW Public Health Act 2010 (No. 127); Primary Health Network information from the Australian Government Department of Health (https://www.health.gov.au/our-work/phn); Cancer Institute NSW from its “What We Do” page (https://www.cancer.nsw.gov.au/what-we-do); Aboriginal Community Controlled Health Services and Aboriginal Medical Services from the Aboriginal Health and Medical Research Council (https://www.ahmrc.org.au/) and NACCHO (https://www.naccho.org.au/); Gastroenterological Society of Australia from its “About Us” section (https://www.gesa.org.au/about/about-us/); ASHM from https://ashm.org.au/about/; Hepatitis NSW from https://www.hep.org.au/about-us/; ACON from https://www.acon.org.au/about-acon/; and the NSW Users and AIDS Association from https://nuaa.org.au/our-vision/.

**Figure 1:**
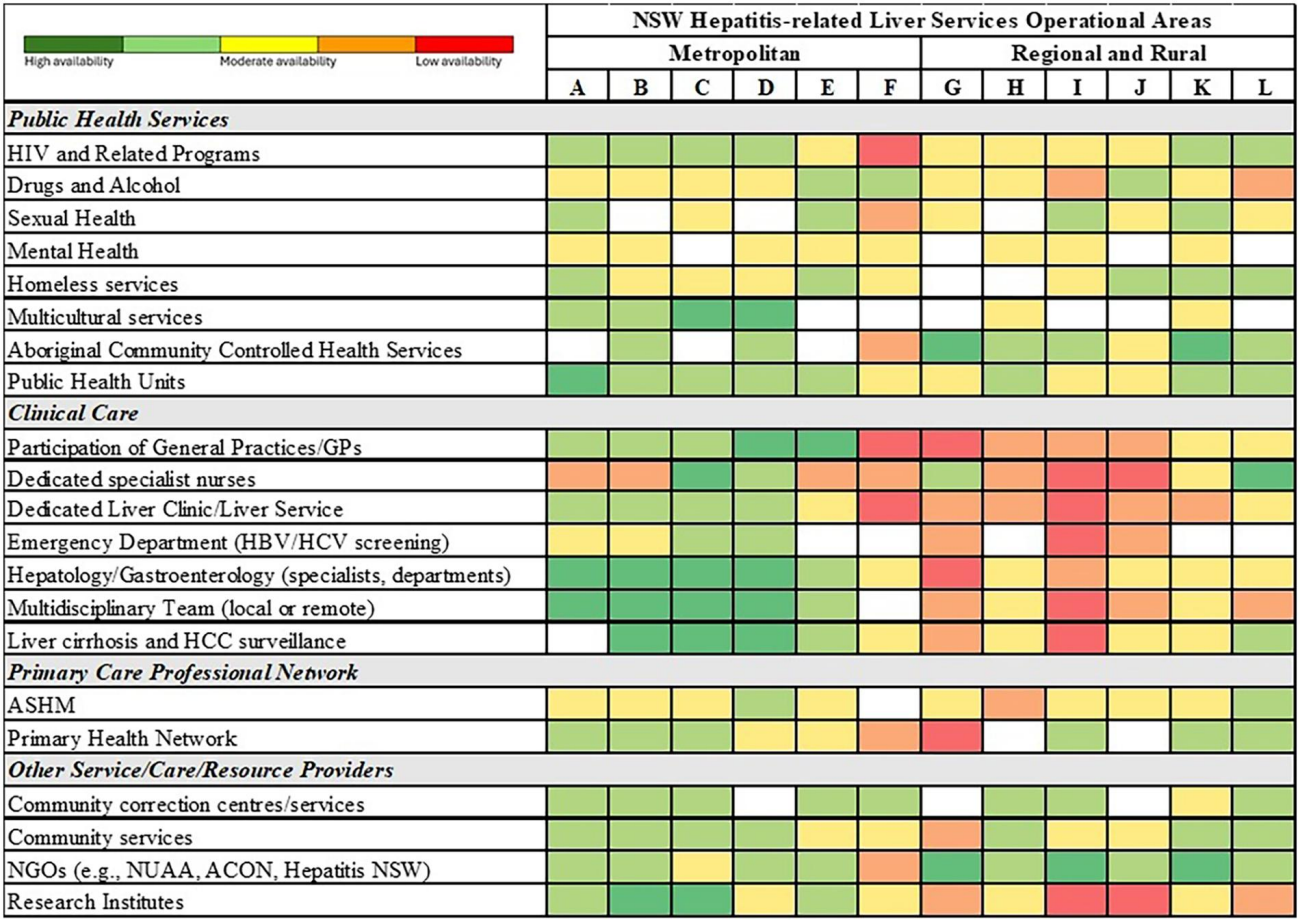
Key stakeholder and resource availability for hepatitis-related liver care in NSW. The colour gradient represents the reported varying levels of resource availability for hepatitis-HCC services, compared to the other operational areas within the same category: dark green—high availability; light green—moderate to high availability; yellow—moderate availability; orange—moderate to low availability; red—low availability. Blank cells—relevant information was not available or not relevant. Areas G, I, L consist of amalgamated services across two LHDs. All information presented is based on interviews conducted with participants in 2023 and 2024. HIV, Human Immunodeficiency Virus; GPs, general practitioners; HBV, hepatitis B virus; HCV, hepatitis C virus; HCC, hepatocellular carcinoma; ASHM, Australasian Society for HIV, Viral Hepatitis and Sexual Health Medicine; NGOs, non-govenunent organisations; NUAA, NSW Users and AIDS Association; ACON, AIDS Council of NSW.

In three cases, two LHDs combined resources and collaborated to jointly provide liver cancer prevention and treatment services, effectively creating 12 operational areas across the 15 NSW LHDs (Figures 1A–L). A total of four stakeholder groups and 21 major stakeholder organisations played roles in the design, delivery, and implementation of hepatitis-related liver services across NSW.

### Organisational complexity in HBV/HCV, liver disease and HCC prevention

Key informant interviews revealed significant variation in hepatitis and liver health services across the 15 LHDs, driven by local demographics, socioeconomic conditions, and the availability of staff, funding, and stakeholder partnerships. Broadly, services fell into two complementary arms: (1) Population-Health Initiatives, delivered via PHUs, HARP, Drug and Alcohol services (D&A) and Sexual Health services, and non-government organisations (NGOs), which coordinate prevention campaigns, community education, Point-of-Care testing (PoC) (40), and rapid referral into treatment; and (2) Clinical Liver Services, provided by specialist nurses, hepatologists, gastroenterologists, and other specialists working in hospitals and outpatient clinics, which oversee antiviral therapy, long-term case management, and six-monthly HCC surveillance. The degree of integration between these arms varied markedly: Multidisciplinary Teams (MDTs), based mainly in metropolitan tertiary hospitals, managed established liver disease, while rural and regional clinicians accessed MDT expertise through informal, personal networks rather than systemic referral pathways. Dedicated specialist nurses and liver clinics (also called hepatitis or satellite liver clinics) acted as the essential linchpin across both arms, bridging prevention and clinical care by coordinating screening, treatment initiation, surveillance, and patient navigation, yet their distribution, seniority, and funding differed greatly between metropolitan and rural/regional settings (Table 3). Although the role of GPs was talked about as potentially important, all stakeholders identified structural and capacity barriers when engaging with GPs.

**Table 3:** Description of key resources available for liver services across LHDs in NSW.

Resource	Operational areas											
	Metropolitan LHDs: A–F						Regional and rural LHDs: G–L					
	A	B	C	D	E	F	G	H	I	J	K	L
Mobile clinic^a^	Y	Y	Y	NR	Y^b^	NR	Y	Y	N	NR	Y^b^	Y
Outreach clinic^c^	Y	Y	Y	Y	Y	N	Y	Y	N	Y	Y	Y
Dried Blood Spot testing	Y	Y	Y	Y	Y	Y	Y	Y	Y	Y	Y	Y
Point-of-Care testing	Y	Y	Y	NR	NR	Y	Y	Y	Y	Y	Y	Y
FibroScan	Y	Y	Y	Y	Y	Y	Y	Y	Y	NR	Y	Y
Nursing capacity^d^ (number of dedicated nurses or FTE)	2	2	5	3 FTE	2	≥2	4 FTE	1.4 FTE	0.6 FTE	1	2.4 FTE	6
Hepatology/Gastroenterology services (local)^e^	Y	Y	Y	Y	Y	Y	N	N	N	N	Y	N
Multidisciplinary Team	Y	Y	Y	Y	Y	NR	Y^f^	Y^f^	Y^f^	NR	Y	Y^f^
Estimated population (% relative to NSW in 2021)	>10	5–10	>10	>10	<5	>10	<5	5–10	5–10	<5	>10	5–10
Estimated geographical area (% relative to NSW in 2021)	≤5	≤5	≤5	≤5	≤5	≤5	>5	≤5	>5	≤5	>5	≤5

Y, yes; N, no; NR, not reported. FTE, full-time equivalent. All information presented is based on interviews conducted with participants in 2023 and 2024. Population data in 2021 via HealthStats NSW (https://www.healthstats.nsw.gov.au/), Centre for Epidemiology and Evidence, NSW Health. Geographic data via the NSW Ministry of Health website.

a Mobile clinics are staffed and equipped vehicles which travel to communities.

b Mobile clinic was funded by the Needle and Syringe Program (NSP) or the NSW Users and AIDS Association (NUAA).

c Outreach clinics are centres in the community (e.g., Drug and Alcohol services, Sexual Health services, Homelessness services) regularly visited by clinical teams for testing and referral.

d FTE figures are reported when specified; otherwise, the number of dedicated specialist nurses were presented. In metropolitan areas, one service reported an impending nurse retirement, another reported one nurse mainly focused on research and data management, a third employs two HBV nurses (HCV nurse numbers unspecified). One regional service had at least two FTE vacancies in 2023.

e Areas that had no Hepatology/Gastroenterology service (e.g., Hepatology/Gastroenterology Department or specialised hepatologist/gastroenterologist) relied on general gastroenterologists whose services included looking after people with liver disease. The capacity of these services varied e.g., one regional area having access to 0.1 FTE of a specialist (2 days per month for 6 months); another had 0.5 FTE gastroenterologist.

f No local multidisciplinary team (MDT) is available; cases are managed through remote MDTs.

### Care cascade for individuals living with HBV/HCV

Participants reported an ideal care cascade in which individuals who test positive for HBV/HCV, whether in community outreach, general practice, emergency departments, or inpatient and outpatient clinics, where patients should be immediately channelled into coordinated treatment and monitoring programs. However, linkage to care often faltered when diagnosis occurred in the community or in primary care. Outreach efforts delivered by HARP units, public health units, mobile clinics, or at community venues such as homelessness services and local festivals successfully identified HBV/HCV cases but struggled to link them with ongoing care:

“When someone's diagnosed, the most effective way to capture them … is the point of diagnosis. As soon as you refer them on to somebody else, you've almost lost them”. (Participant 41, specialist nurse)

**Figure 2:**
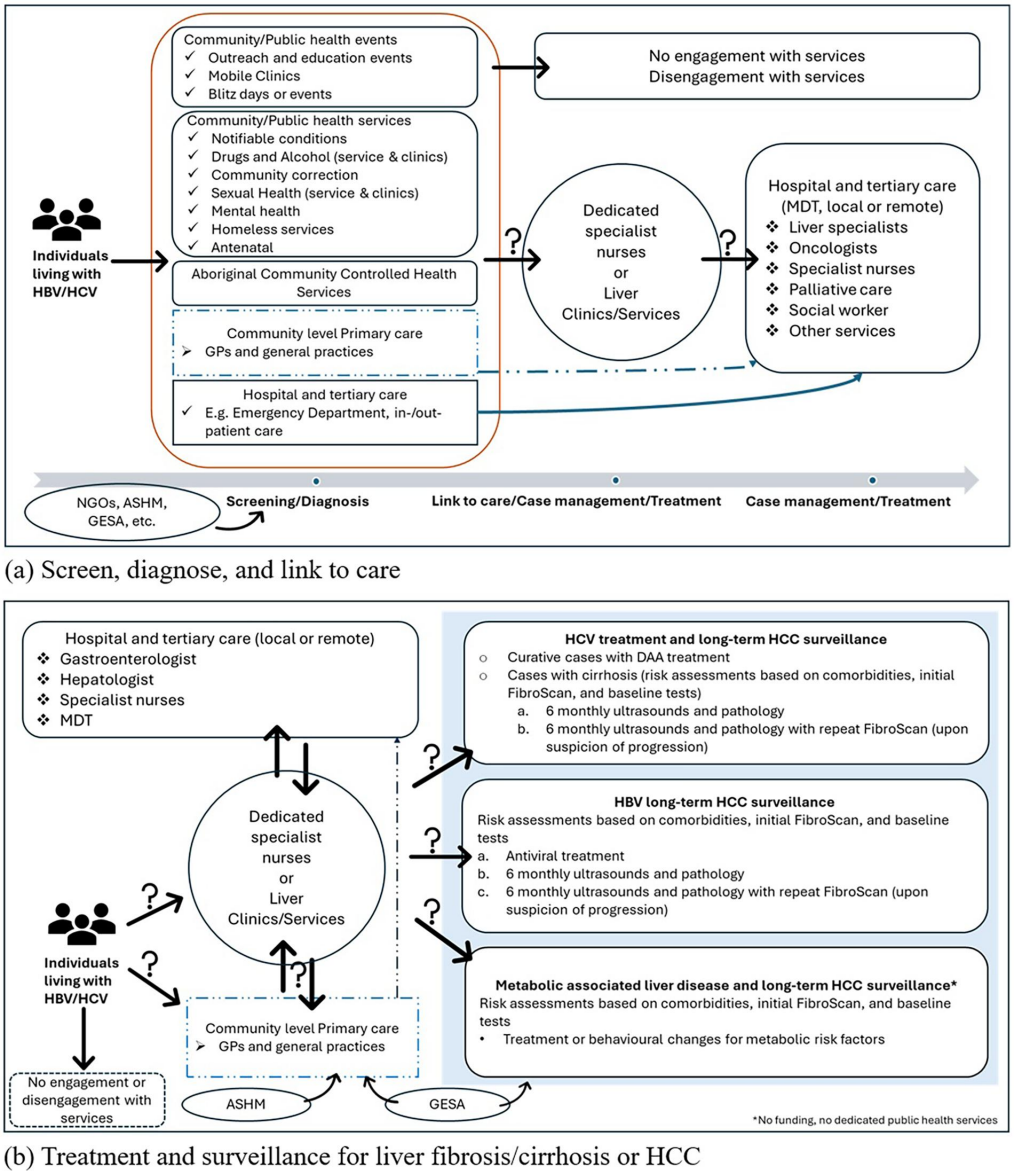
Ideal care journey for individuals living with HBV/HCV and related liver diseases in NSW. (a) Screening, diagnosis, and linkage to care. (b) Treatment and surveillance pathway. MDTs, multidisciplinary teams; NGOs, non-government organisations; ASHM, Australasian Society for HIV, Viral Hepatitis and Sexual Health Medicine; GESA, Gastroenterological Society of Australia; HBV, hepatitis B virus; HCV, hepatitis C virus; HCC, hepatocellular carcinoma; DAA, direct-acting antiviral therapy.

Individuals testing positive through substance use services (including D&A, Needle and Syringe programs, and Opioid Treatment programs), Sexual Health clinics, mental health services, and other community-based services frequently face multiple, parallel referral pathways governed by distinct funding and management structures (Figure 2a). Although all positive serology results are mandatorily recorded in the Notifiable Conditions Information Management System and shared with relevant public health and clinical providers (41), participants noted that notification alone does not ensure engagement with ongoing care. In the ideal model, notification should trigger a no-fee referral to a GP and onward to specialist nurses or liver services for antiviral therapy, six-monthly HCC surveillance, and MDT review when indicated (Figure 2b). In practice, however, significant regional variation in service availability, workforce capacity, and communication channels means that many never complete this ideal journey. The question marks in our care cascade diagrams (Figures 2a,b) highlight persistent gaps and limited integrated referral pathways that bridge community screening with sustained specialist follow-up.

### Care variation, inequalities, health workforce and resource shortages

The scenarios presented in Figure 2, are considerably more complex in practice due to variations in local epidemiology and funding arrangements. Not all LHDs had access to dedicated liver services or specialist nurses. Where such services or nurses existed, they were constrained by funding models available to Medicare-covered individuals only, leaving recent or temporary immigrants, and people without a stable residential address with limited access to free care:

“…people living with HBV who are predominantly seasonal workers and migrants … they become displaced and don't have Medicare … they end up not commencing on hepatitis B treatment and also not linked into any monitoring or ongoing surveillance at all”. (Participant 17, specialist nurse)

Participants also reported that funding streams narrowly focused only on HCV also created inequities, where HBV related disease continued to be:

“…in a grey area of who's doing that and how we're going to approach … that”. (Participant 8, specialist nurse)

Due to the HCV-centred funding, service delivery and access hinge on patients presenting with specific risk factors and engaging with aligned services:

“You've gotta hope. Maybe, maybe they're on methadone, and maybe you can use your methadone case management. Maybe they're homeless and you can use a homeless case manager, but there's very little ring-fence support for actually identifying people who the primary issue is hepatitis, and the new case management around all the things going on to get them on to treatment”. (Participant 7, Sexual Health service staff)

By focusing funding streams narrowly on hepatitis, dedicated liver services are forced to exclude cases where liver damage stems from other causes:

“… everything is purely viral [hepatitis], that is the problem, we don't have a full liver service. We screen a lot of viral for liver cancer and we currently can't do [metabolic-associated liver disease] because of the way we're funded”. (Participant 31, gastroenterologist/hepatologist)

This creates inequities in access to care, felt particularly acutely in rural/regional areas, where healthcare workforce capacity remains critically limited, with shortages of trained specialist nurses, hepatologists, and gastroenterologists. Shortages of GPs in rural/regional areas were also highlighted, and the increasing trend of GPs billing out-of-pocket fees in addition to Medicare reimbursement has made care unaffordable for vulnerable populations.

All participants reported it was difficult to reach, engage, and retain high-risk groups in ongoing care, and resource limitations prevented them from actively following up with individuals from these hard-to-reach groups: people from culturally and linguistically diverse (CALD) backgrounds, those with mental health disorders, people who use drugs and alcohol, individuals with unstable housing, and those living in rural/regional areas. Participants noted that, in rural areas, individuals with HBV/HCV-related HCC often presented with advanced HCC, at a stage when treatment options were already limited.

At least five HCPs expressed concerns that even when people initially engaged with the six-monthly HCC surveillance, many soon disengaged, and there were limited resources to help with re-engagement, as described by a hepatologist working in a metropolitan area, with a socioeconomically disadvantaged population:

“…we see so many people—it doesn't even surprise us anymore, “I used to come here, and I used to do all these tests and then I stopped coming… I'm gonna die now [because of HCC]”. …we have one nurse and a bit [hepatology], so the resources are thin …people just drop off because they don't see value in it”. (Participant 34, hepatologist)

### Main models of care functioning across NSW

Through interviews with 42 participants, **three overarching service models emerged**, each reflecting unique geographic, demographic, and resource contexts yet sharing inherent complexity and variation. Rich picture diagrams were developed to capture their defining features, key care pathways, and points of fracture.

#### Model 1: Rural service model

In sparsely populated rural LHDs, some covering land areas larger than entire European countries, chronic underfunding, minimal infrastructure, and a transient workforce shaped a service model that relied heavily on primary prevention initiatives and mobile outreach. HCV screening and education were often spearheaded by collaborations among HARP units, D&A services, Aboriginal Community Controlled Health Services (ACCHS)/Aboriginal Medical Services (AMS), specialist nurses, and peer workers, supplemented by NGO resources [e.g., NSW Users and AIDS Association (NUAA), AIDS Council of NSW (ACON), Hepatitis NSW]. Mobile clinics equipped with Point-of-Care testing and FibroScan® (42) technology traversed remote regions, while partnerships with Needle and Syringe and Opioid Treatment programs extended reach into community pharmacies and D&A clinics. Despite these efforts, tracking chronic HBV/HCV cases remained ad-hoc, managed through standalone Excel spreadsheets or generic electronic medical record (eMR) reminders that are neither standardised nor fit for purpose. Operational leadership is often disconnected from strategic decision-making:

“…hep[atitis] C and even more so hep[atitis] B have always been the poor cousins … the hospital system … has been quite happy to utilise the hepatitis nurses and increase their scope without increasing their pay, without actually funding those positions or expanding that service…” (Participant 16, HARP staff member)

Treatment initiation, follow-up, and HCC surveillance depended on nurse-led coordination and periodic “fly-in fly-out” specialist visits every three to six months, while local GPs and hepatologists were scarce:

“…once these towns don't have a GP, we're reliant on locums … the primary care provision isn't as good as it should be … So, it all falls apart pretty quickly”. (Participant 27, oncologist)

In this context, specialist nurses developed numerous informal “workarounds”, arranging community engagement by peer and NGO workers and linking patients to the next available outreach clinic:

“[at outreach, our hepatitis nurses] … giving them a number they can call … lined up with the nurse the next time the nurse is doing outreach”. (Participant 16, HARP staff member)

#### Model 2: Integrated care model

In more densely populated coastal and regional LHDs, characterised by ageing populations and accessible specialist services, a second model integrated prevention, treatment, and monitoring within a single framework. HARP units led community education, opportunistic screening, and outreach “blitzes”, while dedicated specialist nurses coordinated treatment initiation and ongoing care in collaboration with PHNs, ASHM, justice and correctional services, D&A services, Sexual Health clinics, community organisations, and ACCHS/AMS. Major regional hospitals often enriched this model with part-time dietitians and social workers, fostering truly interdisciplinary approaches. However, capacity constraints were acute:

“…we have significant patient risk and morbidity and mortality … we have 1,700 people waiting developing cancers…” (Participant 31, gastroenterologist/hepatologist)

Strategic and operational leadership remain misaligned, perpetuating cycles of recruitment and turnover as trained staff depart for better working conditions and higher remuneration elsewhere. One regional specialist described being unable to activate underutilised equipment due to a lack of sustainable funding or Medicare billing codes:

“…I can't develop a service … until it becomes Medicare billable … I have no way of screening people … despite having $440,000 of equipment in the corridor … aging”. (Participant 31, gastroenterologist/hepatologist)

Telehealth and remote specialist networks sometimes extended beyond NSW borders, mitigating local shortages, but they required additional patient transport, funding, and advocacy to overcome stigma and access barriers. As with the rural model, narrow HCV-specific funding and GP shortages exacerbated care gaps and undermined treatment adherence.

#### Model 3: Integrated multidisciplinary partnership model

In metropolitan centres, well-established collaborative networks supported a third model that spanned prevention in the community through to tertiary care. Public health and HARP units coordinated targeted education and mobile testing, linking identified patients to tertiary liver services staffed by specialist nurses and MDTs with formal HCC surveillance programs. Screening was embedded across D&A programs, Sexual Health, mental health, ACCHS/AMS, and diabetes services. In hospital settings, pilot programs in selected EDs implemented large-scale hepatitis screening and linkage to care pathways (43) have further expanded opportunities for case finding:

“People coming through emergency departments … we can “catch” them while they're here focused on their health, got a phone number that might be connected … [we're] able to wrap around services for people who are homeless and have chaotic lifestyles, able to support them all”. (Participant 26, specialist nurse)

PHNs and ASHM enhanced primary care capacity through GP education initiatives [e.g., REACH-B (44)]. Tertiary clinical liver teams provided direct access platforms [e.g., Project ECHO (45, 46)] that enabled primary healthcare providers to consult with specialist networks:

“[Project] ECHO is essentially a way for us to [tele-]mentor general practitioners … non-gastroenterological specialist, specialist units like Drug and Alcohol and Sexual Health … we provide rapid feedback on their cases, so that they can own the case … within the first five years of the ECHO program, we had five hundred additional treatments.” (Participant 24, gastroenterologist/hepatologist)

**Figure 3:**
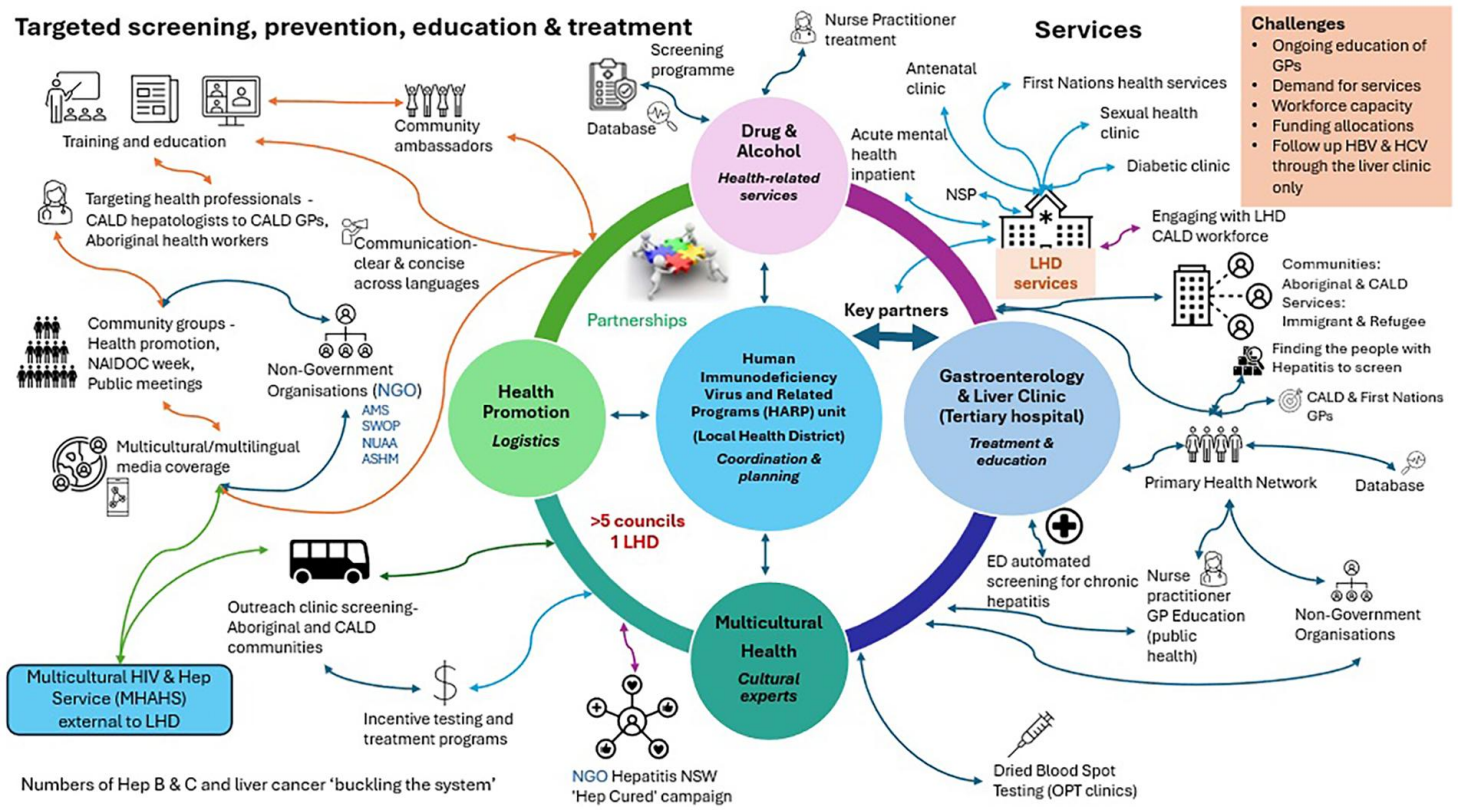
An example of an integrated partnership model in a metropolitan LHD. This rich picture aim to capture the key components and linkages operating in real-life service models (2023-2024), however, due to the complexity, diversity, and change over time, not all details may be captured. CALD, Culturally and Linguistically Diverse groups; HARP, Human Immunodeficiency Virus and Related Programs unit; GP, General Practitioner; NAIDOC, National Aborigines and Islanders Day Observance Committee; LHD, Local Health District; ED, Emergency Department; HBV, hepatitis B; HCV, hepatitis C; AMS, Aboriginal Medical Services; SWOP, Sex Workers Outreach Project; NUAA, NSW Users and AIDS Association; ASHM, Australasian Society for HIV, Viral Hepatitis and Sexual Health Medicine.

Together, these efforts enhanced capacity and confidence of GPs and enabled primary care-led screening, case management, and treatment for hepatitis and chronic liver disease. These metropolitan hubs also extended clinical support to regional and rural LHDs (Figure 3). However, resource constraints were reported as a key barrier. Despite the presence of specialist liver services, specialist capacity was insufficient for the large populations they served. Remote and rural communities also relied on these metropolitan MDTs, further stretching available capacity. Workforce capacity did not reflect service demand or population need, and limited equipment and the absence of integrated recall systems further hindered the ability to achieve comprehensive population coverage:

“…we've got very limited specialist services and they're very, very stretched … the regular six-month screening is not done at six months … people end up in Emergency [Department]”. (Participant 15, D&A service staff member)

Reliance on research grants to supplement nursing positions and trial-based equipment acquisition (e.g., PoC and FibroScan®) underscores the unsustainability of current funding models. One HARP staff member reflected on the restrictive eligibility criteria of pilot programs:

“…if it was just available to everyone, we could have utilised it much more easily, but there were a lot of restrictions”. (Participant 9, HARP staff member)

Together, these three models illustrate the adaptive strategies and persistent vulnerabilities of hepatitis and HCC care throughout NSW, highlighting the need for cohesive funding, workforce development, and interoperable systems to bridge care gaps and ensure equitable access at every level of the health system.

## Discussion

This study represents the first jurisdiction-wide mapping of HBV/HCV and HCC services across NSW, drawing on interviews with 42 healthcare professionals and public health staff from all 15 LHDs. The adoption, delivery, and sustainability of liver health services in NSW are shaped by multilevel structural, and organisational factors. Amid complex and fragmented service structures, we observed notable innovations, such as metropolitan liver cancer MDTs providing tele-mentoring to rural sites, and nurse-led outreach clinics. However, we also identified five interlocking system-level challenges that undermine care continuity and equity of access: (1) inadequate and insecure workforce; (2) fragmented care pathways; (3) misaligned funding streams; (4) ad-hoc data and information systems; (5) stigma and limited community awareness.

### Challenge 1: inadequate and insecure workforce

A workforce with the right skills, capabilities and experience to deliver person-centred care is central to the successful delivery of liver health services (29, 30, 45–48). However, chronic shortages of appropriately trained staff were evident (49–51) across all LHDs, especially in regional and rural areas. Each LHD employs, on average, two specialist nurses, many working part-time on short-term contracts; medical specialists faced funding constraints while balancing significant administrative loads with clinical work. This created capacity constraints, necessitating ad-hoc workarounds and reliance on NGOs or research staff and equipment to sustain essential services. Similarly to other workforce constrained contexts (52), there were flow-on impacts including long patient wait times and limited capacity to re-engage patients lost to follow-up. In some regions, funding for specialist nurses, hepatologists, or gastroenterologists were renewed every six to twelve months, creating job insecurity, staff burnout and high turnover. Lack of GPs (53), specialist nurses, hepatologists, gastroenterologists, and locally accredited antiviral prescribers was particularly evident in rural areas. Addressing these gaps would require the establishment of multi-year, fully funded nursing and medical clinician roles with protected clinical and professional development time (54, 55), together with programs such as rural—metropolitan rotation (56) and regular tele-mentoring clinics to build local prescribing capacity (44–46) and stabilise the workforce.

### Challenge 2: fragmented care pathways

Fragmentation between population health programs and clinical liver services disrupted the continuum of care from primary prevention to long-term surveillance. Our findings showed that screening and early intervention programs are largely delivered through local short-term projects or research trials, with limited collaboration among LHDs. In the absence of a centralised HCC surveillance program, adherence to six-monthly disease monitoring remained suboptimal, especially in primary care settings and in rural areas (57). Despite efforts from ASHM and PHNs to upskill GPs, workforce shortages and the low priority placed by PHNs on HBV/HCV testing and treatment created capacity gaps. The recent boost to Medicare funding across Australia may improve access to GP care (58), however, there are no specific incentives to support care integration and continuity (45, 46). To create a seamless continuum, statewide care pathways needed to be co-designed with patients, nurses, PHUs, HARPs, GPs, liver and cancer specialists.

Project ECHO communities of practice addressed fragmented care pathways by upskilling primary care providers to build capacity and confidence among GPs to care for patients on the hepatitis-HCC continuum, while being supported by trusted specialists. There are opportunities for Project ECHO to align and incorporate training, support and resources available through the REACH-B program to further build capacity for antiviral prescribing and implement guideline-based monitoring for patients with HBV in primary care (44–46). Nurse-led coordination hubs to manage referrals, follow-up, and shared electronic care plans, could substantially enhance adherence to HCC surveillance (59, 60). This model is already emerging in some LHDs and has potential for broader adoption across liver health services and primary care.

### Challenge 3: misaligned funding streams

The NSW budgets for liver disease services remain primarily tied to legacy HIV prevention programs, and a focus on HCV driven by national funding for HCV treatment, while HBV, metabolic-associated liver disease, and HCC surveillance remain under-resourced. Metabolic-associated liver disease is surpassing viral hepatitis as the leading risk factor for HCC, driven by ageing populations, high rates of obesity, diabetes, and alcohol use (1, 2, 61). The singular focus on hepatitis for primary prevention is no longer adequate to prevent HCC. In addition, national-level policy restricts Medicare billing, increasing out-of-pocket costs charged by GPs (58, 62), further excluding marginalised groups. PoC testing is yet to be nationally adopted, limiting detection of HBV/HCV (63). Routine repeated medical imaging (e.g., ultrasound or MRI) recommended for HCC surveillance, is not fully covered by Medicare (62), forcing individuals with chronic liver disease to pay out-of-pocket costs that many cannot afford. A weighted, epidemiology-based funding model that allocates resources according to disease burden, socioeconomic disadvantage, and remoteness, and the introduction of new Medicare item numbers for PoC diagnostics and routine imaging would eliminate financial barriers, potentially increasing HBV/HCV detection, treatment uptake, and adherence to long-term surveillance to optimise outcomes (64–66).

### Challenge 4: Ad-hoc data and information systems

In NSW, the Notifiable Conditions Information Management System and the NSW Cancer Registry capture diagnostic events but lack longitudinal tracking or automated recalls. In the absence of a centralised patient-tracking system, clinicians resort to standalone spreadsheets or local electronic medical record reminders. This hinders information sharing among LHDs, and individuals often drop out of monitoring after relocating to another region. The ASHM “REACH-B” program, designed to break down siloed information systems and enhance GPs’ capacity for screening and case management, holds promise if it is taken up by GPs (44). The NSW single digital patient record (SDPR) (67), planned for implementation by 2028, promises broader interoperability enabling patient tracking and recall to support retention in HCC surveillance programs. Disappointingly, the SDPR, as currently planned, excludes many community and NGO-delivered services, although this may be considered in the future. Developing a statewide, secure, real-time liver health registry, integrated with the SDPR and compliant with Fast Healthcare Interoperability Resources (HL7 FHIR) standards (68), alongside shared dashboards and automated reminder systems for hospitals, GPs, and allied health providers, would enable statewide surveillance, reduce time-to-treatment initiation, and minimise patient disengagement.

### Challenge 5: stigma and limited community awareness

Participants in our study highlighted widespread gaps in public knowledge of hepatitis, and long-term risks of HCC. Traditional public health messaging framed hepatitis and HCC risk around HIV, injecting drug use, risky sexual practices, or incarceration, alienating older adults, migrants, and those with metabolic risk factors. A shift in narrative towards “Liver Health” is needed to destigmatise liver disease, and foster open, non-judgmental conversations in health and community settings (61, 69).

Efforts to increase community awareness could include co-developing culturally responsive campaigns with Aboriginal Community Controlled Health Organisations, CALD organisations and media outlets, and faith-based groups to improve reach. Training and resourcing “Liver Champions” such as GPs, community nurses, peer workers and educators to deliver targeted outreach through pop-up clinics and education sessions, offers a promising strategy (70).

Viewed through the CFIR framework domains (36), the challenges identified in this study align closely with the multilevel factors that shape the adoption, delivery, and sustainability of liver health services in real-world settings. Ad-hoc data and information systems constrained innovation, reducing the usability and adaptability of integrated care models. Stigma and limited community awareness reflected critical outer-setting barriers that weaken engagement and delay timely access to care for people living with chronic liver disease. Inadequate and insecure workforce capacity reflected inner-setting constraints and the characteristics of individuals, where shortages, turnover, and limited protected time undermined liver health services’ organisational readiness for implementation. The outer-setting issues, including misaligned policies, funding that was not fit-for-purpose, and limited communication and cross-sectoral partnerships, led to care pathway fragmentation and limited care continuity. Participants also noted limited service planning capacity, coordination, and opportunities to scale up effective local innovations. Together, these interconnected barriers illustrated the system-level constraints that must be addressed to achieve sustainable, integrated liver care across NSW.

### Strengths and limitations

Our study fills a critical knowledge gap about the integration of primary prevention programs for hepatitis with care pathways for liver disease. A key strength of our study is its inclusiveness: engaging frontline workforce from all 15 LHDs, triangulating inductive thematic analysis with rich picture visualisations, and conducting member checking to ensure credibility. The multi-stakeholder steering group further enhanced methodological rigour and practical relevance. However, reliance on self-reported experiences and practices may introduce recall and social desirability bias, and the exclusion of incarcerated populations, wider policymaker and peer worker perspectives may underrepresent certain experiences. While this study focused on understanding service structures and implementation issues from a system perspective, a key limitation is the absence of the lived experience voice and expertise. Although people with lived experience of chronic liver disease were part of our steering committee and helped guide the study, their perspectives contributed to the design and interpretation rather than being directly represented in the dataset. Incorporating lived experience insights is essential and will need to form the basis of future work, where these perspectives may reveal different barriers and priorities for improving integrated liver care (71). Finally, the context-specific nature of our findings may limit direct generalisability to other jurisdictions.

## Conclusion

The main NSW liver health policy documents set ambitious goals, such as eliminating HBV and HCV as public health concern by 2030, universal screening, seamless treatment pathways, and co-designed, person-centred, and integrated prevention and care (29, 30, 48, 72). Advancing these strategies requires robust cross-sector organisational systems, equitable resource allocation, targeted workforce development and meaningful community partnerships.

By addressing these interwoven challenges through a stable and well-funded workforce, standardised and co-designed care pathways, equitable and epidemiology-driven funding, interoperable data infrastructures, and destigmatised public messaging, NSW Health could bridge policy and practice and transform isolated innovations into a cohesive, equitable liver health continuum. Advancing this work will require longitudinal evaluation of service integration initiatives, together with the deeper inclusion of lived experience and policy-maker perspectives and partnerships, to inform more responsive and effective service co-design and improvement. Such integrated reforms will accelerate progress toward WHO hepatitis elimination targets and offer a scalable blueprint for comprehensive liver care around the world.

## Data Availability

The datasets presented in this article are not readily available because the data generated and analysed are subject to confidentiality commitments made to participants during the informed-consent process. Requests to access the datasets should be directed to Yvonne Zurynski, yvonne.zurynski@mq.edu.au.
