# Supplementary material for: Healthcare system complexity of hepatitis B and C related liver disease: service mapping in New South Wales, Australia
Source: Front Health Serv. 2026 Jun 25;6:1842589. doi: 10.3389/frhs.2026.1842589 (PMC13345870; doi:10.3389/frhs.2026.1842589)
Supplement: Supplementary file 1 [file Table1.pdf]

Appendix file 1

Service mapping for hepatitis-HCC care in NSW, interview topic guide (*Public Health staff*)

1. Tell me about your HARP (Public Health/D&A/Sexual Health, etc.) Unit and what you do
2. What are the priorities for your unit over the next 12 months?
3. What programs do you run for primary prevention of HBV and HCV? For example, screening programs, community outreach, education, or supported referral?

(**Prompts:** For HBV/HCV **screening programs** - If you have HBV and HCV screening programs in your LHD, then who is the target population, who delivers the services and how, and who provides the funding? Any examples you could share? For **education programs** on HBV/HCV - If you have HBV and HCV related education programs in your LHD, then who is the target population, who deliver the programs, and who provide the funding? Any examples you could think of?)

4. What strategies/programs does your unit implement to enhance HBV and HCV testing, linkage to care, and treatment uptake? For example, facilitated referral for HBV and HCV assessment, scheduling of specialist appointments, integrated care, drug use, and psychiatric services, case management services?
5. Tell me about the population in your LHD and which groups of people are you particularly concerned about in terms of HBV and HCV prevention and why?
6. Tell me about the resources you have at your disposal to implement HBV and HCV prevention strategies.

(Prompts: what budget do you have for primary prevention of viral hepatitis? Apart from the core budget, do you have other sources of funding for specific programs related to HBV and HCV prevention?)

7. What resources do you wish you had, but don't have at the moment?
8. How do you monitor or evaluate the success of your prevention programs?

(**Prompts**: do you produce any regular reports; are they publicly available? How often do you evaluate your HBV and HBC prevention programs?)

9. When you evaluate your programs, do you ever consider longer term outcomes, including the development of liver cirrhosis and liver cancer?
10. Tell me about the key partnerships that make your work in HBV and HCV prevention possible?

(Prompt: How do you find partnering with NGOs and community groups? (ask if relevant)

11. What are your opinions about the latest strategies released by the Ministry?

12. Do you believe your unit has the capability to implement these strategies in your local region?

(Or are you already implementing some of the strategies covered?)

13. Is there anything else you would like to add, or do you have suggestions on improving primary prevention of HBV and HCV and liver cancer in you region

Appendix file 2

Service mapping for hepatitis-HCC care in NSW, interview topic guide (*Healthcare professionals*)

1. Tell me about your healthcare institution (e.g., hospital, liver centre, liver clinic, PHN…) and what you do
2. What are the priorities for your clinic or service over the next 12 months?
3. What programs do you run for prevention of viral hepatitis and liver cancer? For example, antenatal vaccination, screening programs, community outreach, GP education, supported referral, remote prescription?
4. (**Prompts:** For **screening programs** - If you have HBV, HCV, or fibrosis screening programs, who is the target population, who delivers the services and how, and who provides the funding? Any examples you could share? For **education programs** - If you have viral hepatitis or liver cancer related education programs, then who is the target population, who deliver the programs, and who provide the funding? Any examples you could think of? Are the education programs focusing on GP training, or do you provide community education as well?)
5. What strategies/programs does your clinic or service implement to enhance HBV and HCV testing, linkage to care, treatment uptake, and monitoring for fibrosis and cirrhosis? (For example, vaccination, facilitated referral for liver stiffness assessment, scheduling of specialist appointments, integrated care, psychiatric services, and case management services)
6. Tell me about the population in your care and which groups of people are you particularly concerned about in terms of viral hepatitis and liver cancer and why?
7. In your areas, what are the healthcare or referral pathways for patients with chronic viral hepatitis infection, fibrosis, or cirrhosis? What types of liver cancer services are you providing?
8. What challenges have you encountered when delivering or implementing the primary prevention programs? (e.g., patient adherence, GP motivation, collaboration between service providers, etc.)
9. Tell me about the resources you have at your disposal for prevention of viral hepatitis and liver cancer.
10. **(Prompts:** what budget do you have for prevention of viral hepatitis and liver cancer? Apart from the core budget, do you have other sources of funding for specific programs related to the prevention?)
11. Tell me about how do you monitor or evaluate the success of your prevention programs in primary prevention of viral hepatitis, fibrosis and liver cancer?

12. (**Prompts**: do you produce any regular reports; are they publicly available? How often do you evaluate your prevention programs?)
13. Tell me about the key partnerships that make your work in chronic liver disease prevention possible?
14. **(Prompt:** How do you find partnering with LHDs, PHNs, NGOs and community groups? (ask if relevant)
15. Are you aware of the latest viral hepatitis strategies (released by the Ministry), the new NHMRC guidelines and roadmap on liver cancer control (by Cancer Council)?
16. What is your opinion about the implementation of these guidelines? Is your clinic/unit or LHD currently implementing these guidelines? If so, how?
17. Is there anything else you would like to add, or do you have suggestions on improving prevention of viral hepatitis, chronic liver disease, and liver cancer in you region?
